# "The Hospital" Nursing Mirror

**Published:** 1897-06-26

**Authors:** 


"The Hospital" Nursing Mirror.

Being the Nursing Section of "The Hospital."

[Contributions for this Section of "The Hospital" should be addressed to the Editor, The Hospital, 28 & 29, Southampton Street, Strand, London, W.C., and should have the word "Nursing" plainly written in left-hand top corner of the envelope.]

### News from the Nursing World.

### THE ROYAL RED CROSS.

The Queen has conferred the decoration of the Royal Red Cross upon Nursing Sister Louisa Watson Tulloh, of the Army Nursing Service, in recognition of her services in tending the sick and wounded in Egypt, from 1888 to 1894.

### ROYAL DERBYSHIRE NURSING ASSOCIATION.

Several interesting facts were reported at the annual meeting of the Royal Derbyshire Nursing and Sanitary Association, held at the Victoria Hall, Derby. Both as regards the quality of the nurses' work, the demand for their services, and from a financial point of view the report was satisfactory. This year a specially-designed badge in bronze, with name bar attached, has been sanctioned by the committee to be worn by the nurses as a means of easy identification, and a new form of certificate, with an appropriate design, is in future to be presented to the nurses on completing a term of service. In bonuses and extra fees the board have been able to divide among the nurses a sum of £173 17s. 4d., and by way of marking the Diamond Jubilee have also voted to each nurse on the staff a special "Jubilee bonus." The work of the district nurses, of whom there are six, has so much increased that funds are being appealed for to add three more to their number, and a resolution in favour of this extension was adopted at the meeting.

### A PLEA FOR MALE NURSES.

A writer to the *Morning Post* lately put forward the case for the male nurse, asking how it is that there are practically no hospitals in England where men have the same opportunities of being trained for nursing as women have. Seeing that it is acknowledged on all sides by those who know anything of the matter that trained and efficient men nurses are much needed, it is certainly extraordinary that England is so far behind America in this respect. "J. H. L.," the correspondent in question, says: "Attempts have been made to induce the authorities of the metropolitan hospitals to give the same training to men to become nurses as they give to women, but without success," and considers the reason to be that the "matrons of British hospitals have put up their backs against the proposal, desiring to keep nursing in the hands of their own sex, and the hospital managers have been weak enough to yield to them." He thinks that now, when rich people are giving lavishly to hospitals, is the time for these benefactors to bring their influence to bear on the authorities to rectify this condition of things. We hardly think that English matrons deserve this imputation of narrow-minded prejudice against a system which should lead to an invaluable extension of the benefits of trained nursing; committees are probably more to blame in their dislike of a new departure. At one London hospital at least—the National Hospital for the Paralysed and Epileptic in Queen Square—a limited number of men are trained as probationers in the wards under the supervision of the sister, and we understand that the experiment has been successful. It is a very great pity that the example thus set has not as yet been followed elsewhere.

### NURSING THE PLAGUE.

Miss Piggott, who was formerly closely associated with the Victoria Nursing Home at Cheltenham and its work among the poor, is now helping to nurse plague patients in India. The *Sind Gazette* states that immediately the plague hospital was opened at Hyderabad Miss Piggott offered her services to nurse in its wards as a volunteer, and that "the hospital is indebted to her for more than can be expressed in words."

### COMBER DISTRICT NURSING SOCIETY.

At the recent annual meeting of this association, presided over by Lady Helen Stewart, daughter of Lord and Lady Londonderry, a delightful address was given by Lady Margaret Hamilton, in which she spoke of the inestimable advantages a district derived from the presence in it of a trained nurse—"a woman, properly qualified and trained, planted down in the midst of the people." All who have seen for themselves the immense power for good which may be wielded by these trained and qualified women in the course of their daily work will agree with Lady Margaret when she described the district nurse as the most successful of all agents for the betterment of the poor in their own homes. "The district nurse's personality," she said, "operates as no impersonal suggestion or institution can; she is there to be referred to, to be taken counsel with, to be the friend of all; and her influence is felt, her advice taken by many who cannot accurately be described as sick." Lady Margaret went on to impress on her hearers an important fact which is being much overlooked in many places just now, and that is the imperative need that the district nurse, if she is adequately to fulfil her mission, shall be well and thoroughly trained, and that in hospital as well as in district work. Much appreciation of Nurse Fenton's devoted work was shown by the various speakers, Dr. Henry saying that he gladly noted a marked improvement in sanitary matters in Comber, an improvement which he attributed to Nurse Fenton's influence.

### THE NURSES' NEEDLEWORK GUILD.

A needlework guild has lately been started by a few nurses with the object of collecting women's and children's clothing, the result of odd moments' work, to be distributed amongst hospitals, homes, and charities. The expenses incurred by postage, printing, &c., are to be met by an annual subscription of sixpence from members and one shilling from associates, ladies other than nurses being invited to join in the latter capacity. Nurse members are to contribute one garment yearly, and associates two. Nurses Crouch, Goodin, Lewis, and Stutter, of the Nurses' Co-operation, New Cavendish Street, Nurse Edith Theobald, St. Thomas's

Hospital, and Nurse A. M. Theobald, 4, Bolsover Street, form the working committee, and they hope many of their fellow nurses will join the guild and do what they can to make it a success. The demand upon time and purse is but small, so that Nurse A. M. Theobald, who is the honorary secretary, ought to receive many applications from would-be members. She will be delighted to give full particulars to all inquirers.

### BIRKENHEAD AND THE JUBILEE.

A SCHEME has been initiated by the Mayor of Birkenhead in commemoration of the Queen's reign which will include the addition of a new wing to the Birkenhead Borough Hospital, the establishment of a home for trained nurses, and the provision of free skilled nursing for the sick poor of the town in their own homes. It is wisely proposed to provide nurses on a graduated scale for that other numerous class of persons who can afford a small payment, and to these fees according to their means will be charged. Of the £6,000 required to carry this scheme through, £3,775 has been already promised.

### DISTRICT NURSING IN SUFFOLK.

AT a recent meeting of the Suffolk Nursing Association, held at Ipswich, Miss Gertrude Barnadiston, the secretary, reported that four districts had now been formed, at Stowlangtoft, Badwell, Walsham, and Horringer, and placed in charge of trained district nurses; that six additional nurses were now being trained; and that by the coming autumn at least ten districts would be in full working order.

### THE NURSES' HOSTEL COMPANY (LIMITED).

WE announced some time ago that the conversion of the Nurses' Hostel in Percy Street into a limited liability company would soon be carried out, and we have now received a copy of the prospectus. The Nurses' Hostel, it will be remembered, was opened in 1889 by Miss Catherine J. Wood, and as a home for private nurses, and a hotel for those who needed temporary accommodation, has provided for a real want among nurses, and for seven years has paid its way. When, owing to the expiry of the lease of the house, a change became necessary, a site was secured in Francis Street, Tottenham Court Road, and new buildings, specially designed, erected thereon, with accommodation for fifty nurses. The directors of the new company, which starts with a capital of £10,000, in 1,000 shares, are General Sir Allen Bayard Johnson, K.C.B., Mr. Charles S. Roundell, Miss Mary F. E. Hope, Miss Sarah Clegg, and Miss Catherine J. Wood, the latter joining the board after the allotment of shares. The directors have arranged to take over Miss Wood's interest in the undertaking, and have secured her services as managing director.

### PAUPER NURSING IN IRELAND.

THE cruelty and barbarity of the present system of pauper nursing in Irish workhouses are forcibly stated in a report just issued by Dr. Joseph Smyth, of Naas. The authenticated stories from various unions of the blackmailing and neglect inflicted on the unfortunate occupants of the "sick wards" by these pauper attendants, who are generally women of bad character, "damaged," as the medical officer of Omagh Union, remarks, "mentally, morally, and physically," form a terrible indictment against the boasted humanity of this civilised age. The only way to get these ghastly abuses redressed is to thoroughly arouse public feeling on the matter, and the *Irish Times* and *Irish Independent* are doing well by speaking out strongly. The latter paper says: "It is notorious that the system of pauper nursing is rotten to the core, and we hold that the Local Government Board are seriously at fault in not having taken some steps long ago to reform what they must well know to be an utterly indefensible state of things. The general public cannot, however, be aware of the facts, or there would be such an outcry as would soon sweep away the entire system, and silence anybody inclined to defend it from economical or other motives." Where the nuns have undertaken the nursing things are a little better, and these good women have worked well, but they are not trained nurses, nor are they, as a rule, allowed to undertake any night work. It is a well-known fact that in many of these workhouses the sick and aged are left locked up all night without a light, and that it is no infrequent occurrence to find one of them dead on the floor in the morning. Yet, what is being done to remedy a condition of things which would disgrace a country of heathens and savages?

### HASLEMERE NURSING ASSOCIATION.

THE Haslemere Nursing Association held its first annual meeting early in June. It was reported that the association had received a generous offer from Mr. Penfold to provide a site and build thereon at his own cost a house suitable for the needs of the association, on condition that the association would be of a permanent character. A resolution gratefully accepting this offer was put to the meeting and carried.

### SHORT ITEMS

AFTER the annual meeting at "Friedenheim," the Home of Peace for the Dying at Swiss Cottage, on June 12th, at which Canon McCormick took the chair, those present were invited to inspect the Home. Tea and coffee were served in the grounds at half-past four.—It is proposed to establish a Nursing Institute at Wednesbury, and a meeting in support of the scheme, convened by the Mayor, was well attended.—The fund which is being raised in Argyllshire in aid of the "Queen's Nurses" is to be used exclusively for the needs of the county, and not sent to the central fund.—A district nurse is to be established at the Mumbles in commemoration of the reign of Queen Victoria.—We are glad to hear that Sir S. Bancroft's reading of the "Christmas Carol" at the Imperial Institute, in aid of the Colonial Nursing Association, realised £250.—A number of schemes for celebrating the Queen's Diamond Jubilee were put to the vote at a general meeting held the other day at Stoke Newington, with the result that it was decided to raise funds to maintain a parish nurse.—The Duchess of Sutherland has been energetically advocating the nursing scheme in which she is interested throughout Sutherlandshire, travelling through the remoter parts of the county, and giving addresses at various places. The Duchess everywhere received a warm welcome.—A district nurse is to be established at Denton, Cheshire, in commemoration of "the longest reign."—We are glad to hear that the Matron of Caterham Asylum has instituted classes in elementary nursing. At a recent examination Nurses Florence Johnson, Sophia Cox, Elizabeth Fyfe, and Rebecca Gordon Stuart sent in really very good papers. We congratulate the matron, as this is a move in the right direction, and other matrons might follow suit.

### Pharmacy and Dispensing for Nurses.

By C. J. S. Thompson.

### XV.—SUPPOSITORIES, PESSARIES, BOUGIES, PLASTERS, AND BLISTERS.

Suppositories are used for the administration of drugs per rectum, and consist of some active medicinal agent mixed with a solid base, such as oil of theobroma, stearine, or soap and starch, that melt about the temperature of the body. They are moulded or formed into the shape of a small cone. The moulds generally used are made of metal, and can be divided into two parts, which are held together by a screw.

In making suppositories, the chief art lies in properly incorporating the medicinal ingredients with the base, and transferring the mixture at the right time to the moulds.

Oil of theobroma, the base most frequently used, is a concrete oil that melts at a temperature between 86 deg. and 95 deg. Fahr. To illustrate the process of making suppositories, we will describe how the the following prescription should be prepared: ℞. Pulv. opii, gr. i.; ext. belladon. alcohol, gr. ii.; ol. theobrom, q.s.; ft. supposit, mitti vi. First weigh sufficient of the theobroma oil to make six suppositories (each of which should weigh fifteen grains), and melt it in a small porcelain dish with gentle heat over a water bath. While this is being done, prepare the moulds by wiping them out with soap liniment, so as to leave a thin coating, which will prevent the suppository adhering. Next weigh the powdered opium and extract of belladonna, and placing them on a slab rub them down to a thin paste with a little of the melted oil by means of a soft spatula. When thoroughly mixed, place in the dish with the remainder of the melted oil, and stir the whole thoroughly until it assumes a creamy consistence and will only just pour; then, still stirring, rapidly transfer to the moulds, and allow to stand in a cool place till set.

Another method of mixing when the active ingredients are in the form of powder, is to melt the base in a wide-mouthed bottle and add the other ingredients; cork, and shake well till incorporated, then pour into the moulds. The ordinary extracts of the Pharmacopœia not prepared with alcohol, may be easily mixed with the base if first rubbed down on a slab with a few drops of boiling water. Substances of a crystal line nature, such as bromide of potassium, chloral hydrate, or tannic acid, must be reduced to a fine powder before being mixed with the base. Iodoform should not be heated in the base, but mixed on a slab, after the latter has been melted. Heat must also be avoided when chloral hydrate is employed. Iodide of lead, oxide of zinc, and other heavy ingredients should be rubbed to a smooth paste with a little of the melted oil on a slab, and not transferred to the moulds until just before setting, as they are apt to settle into a hard mass at the apex of the cone.

Glyco-gelatine is largely used as a basis for suppositories, which when not medicated are popularly known as "glycerine suppositories." The base may be prepared as follows: Take of fine gelatine, ʒ vi.; glycerine, ℥iss.; water, ʒ x. Moisten the gelatine first with a little water, then add the glycerine and the remainder of the water, and heat over a water-bath till dissolved. The moulds should be oiled or painted with soap liniment.

### Pessaries and Bougies.

Pessaries are prepared in the same manner as suppositories, only a larger quantity of material is used. They are usually made to weigh 60 or 75 grains each, and run into moulds of suitable capacity.

Medicated bougies are made to weigh 20 grains each, and should be about two and a half inches in length, gradually tapering to a point.

Nasal bougies are usually prepared with a glyco-gelatine base, and are introduced into one of the nasal passages for treatment of chronic affections of the nases. They should be about three inches long, and a quarter of an inch in diameter at the base, the whole gradually tapering towards the apex.

### Plasters and Blisters.

The spreading of a plaster is an operation that requires a considerable amount of practice and skill. It consists in melting, and evenly spreading by means of a warmed spatula, a medicated compound with a base of wax and resin, &c., over a given space of thin leather or other material suitable for the purpose. The plasters met with in dispensing are chiefly those of the Pharmacopœia. They are usually kept in convenient rolls, weighing about half a pound. In melting, a very gentle heat is required, which may be applied by means of the warm spatula, or sufficient of the plaster may be cut off the roll and melted in a small pan. As little heat as possible should be used throughout the process. The material on which plasters are generally spread is the prepared white skin known as "plaster skin." Chamois leather, swansdown, brown holland, calico, &c., are also used for the purpose. Plasters for application to the chest are made heart-shaped, and those for the side or back, oblong or reniform. Those for application to the breast should be made circular. In order to follow out the process in detail, we will take the following prescription as an example: ℞ Emplast. plumbi. 10 × 6. Fiat. emplast. The plaster spatula or iron must first be put to warm. If the ordinary iron is used, it may be heated over a Bunsen burner, or by placing it in the fire for a few minutes. While this is being done cut out the exact pattern of the plaster from a piece of fairly stout paper, leaving a margin of about an inch in depth around the shape. A piece of leather may now be cut, at least an inch and a half each way larger than the dimensions given for the plaster, and several folds of paper placed between it and the counter to form a soft bed to work upon. The paper shape must next be affixed carefully to the leather by means of some weak gum or soft soap, the inner edge being closely pressed to prevent the plaster getting underneath. The plaster having been melted as described above, take the spatula, and with long strokes carefully spread the liquid plaster evenly over the surface of the leather, working from left to right. After allowing it to cool for a few minutes, strip off the paper shape, which should leave a clean sharp edge.

A blister is usually spread on adhesive plaster or thin leather, the shape being cut out of paper, as that for a plaster. Cantharides or blistering plaster, being of a soft nature, the spatula is not necessary, and a sufficient quantity being cut off the roll, it is well worked with the fingers until it is quite plastic and soft. It is now placed on the base to which the shape has been affixed, and spread with the side of the thumb, first around the margins, and then over the centre, until the whole is evenly covered. The surface may be rendered smooth by applying a little olive oil with the thumb if necessary. The shape may now be removed and the margin trimmed. A blister should have a margin of from three-quarters to an inch, according to size. They are made of various shapes, according to the directions of the prescriber.

In the foregoing articles we have only been able to give a bare outline of some of the practical operations which the dispenser is called upon to perform, but we trust they may serve to excite an interest and desire for a closer acquaintance with the art of pharmacy, which has been aptly termed "the handmaid of medicine."

### Post-Graduate Clinics for Nurses.

By a Trained Nurse.

### XIX.—THE PRODUCTION OF REST-CURE PATIENTS.

The weekly instalments of the few last clinics have been insensibly and by slow degrees leading up to the nursing and care of nervous and rest-cure cases. And, since every nursing faculty and every subtlety of the art is called into play by such cases, most of the principles involved in nursing must needs be touched upon in writing about the Weir-Mitchell method of treatment. For instance, the knowledge of dietetics necessary in nerve cases is a most essential part of a nurse's curriculum, since every form of gastric and hepatic trouble and derangement is met with in patients for whom rest-cure is prescribed. If I were asked as to an ideal method of training a nurse, I think I would suggest that her first year should be spent among sick infants and children. She might then take six or nine months work with rest-cure patients, and would thus be magnificently equipped for further training in the surgical and medical wards. While working among the children she would learn habits of observation difficult to gain so well in any other way, and her work in the rest-cure wards would furnish her with self-control, an insight into psychological nursing, and a knowledge of the marvellous bearing that mental and moral conditions have upon health. These important factors are, it seems to me, well-nigh impossible to acquire in any other branches of nursing. The system of "rest-cure" is popularly supposed to have originated in the United States. But antiquity—which is responsible for most of the things called "new"—has a claim to a modified system of rest-cure treatment, and the Orientals have realised long since that rest and Nature may be relied upon to repair much of the wear and tear and the patches made in human health by overwork or wrong usage of faculties. Massage comes entirely from Japan andthe East, and is a practice that has been in vogue for centuries among very primitive peoples. The chief reason why America is so intimately associated in most minds with "rest-cure" is that the condition to which the "nerves" of the Americans have been brought by hurr y and rush has necessitated the perfection and elaboration of an antidote in the shape of rest-cure. One has only to live for a few weeks in an American city to be able thoroughly to realise why rest-cure is a national necessity. For all celebrations of a mercantile, social, and patriotic nature are honoured by noise. Guns' fire "crackers," and every sound-producing kind of ammunition is let off recklessly during large and small town and village rejoicings, and there is no question that every true-born American would dearly love to celebrate all the birthdays, weddings, and christenings occurring in his particular district by the firing of 81-ton and Gatling guns, were these not somewhat rare and expensive playthings. Unquestionably all this noise tells most terribly from one generation to another on the nervous systems of the people. And "rest-cure" homes will need to be built more and more each year until the nation realises the healthfulness of quiet, and that some degree of "dullness" is an essential of daily life. It is curious how many American "improvements" and practical every-day useful time-savers and inventions we are importing to England. And we are at the same time, quite unconsciously, approximating the type of our English women to the American type—and this can hardly be reckoned among the "improvements." "Your English women used to be quiet, comely, and restful to mind and eyes," said a very great American nerve specialist to me not long since. "But when I come to London now, I might almost as well be on Fifth Avenue, for your women are becoming haggard and sallow, jerky in their movements, incessant talkers, and in perpetual motion. They never seem satisfied unless they are using their faculties to the utmost—bicycling, rushing to theatres, shops, and parties; always seeking, seeking, apparently never finding what they seek, but going on like the restless waves of the sea. Looking at the surging crowds of restless women in London," he went on, "I see before me whole generations of neurotics to come. I see children with chorea and epilepsy, and every form of nervous disease. Time was," he concluded, "when I vaunted the English mother as producing as fine a race of men as did ever the Roman matrons. But you won't get efficient motherhood from these hipless, rushing women," he said despondently, as he looked at the bicyclists in Battersea Park. "They're almost as bad as the Americans. I am going to Scotland to see if there are any bonnie women left in the world."

Now things are not quite so bad as he describes, but we are as a nation becoming more nervous, and ordinary illnesses of to-day are unquestionably complicated more than they used to be by "nerves" and irritabilities and restlessness. And the proportion of patients ordered to take rest-cure is undoubtedly growing. So that the method of nursing such patients is more and more necessary to those nurses who wish to keep step with the many branches involved in modern nursing.

Among rest-cure patients must be included a small proportion of victims of overwork, both physical and mental. But a somewhat extended experience in a Weir-Mitchell hospital, under the great specialist's guidance, has led me rather to the conclusion that the greater number of such patients have become eligible for treatment by undue indulgence in the petty worries and irritations of daily life. I do not remember among the patients one woman who needed rest-cure after writing a great book or achieving any large and important work or enterprise. But I can recall dozens who fretted themselves into a dangerous mental and nervous condition through taking house-cleaning and domestic details too seriously. I remember one delicate young woman who became a rest-cure patient in my hospital after attempting to take her medical degree too brilliantly. But there were twenty others who were prostrated by a wearying disposition to fret over trifles. These were women who magnified cake-baking into so important a matter as to lie awake night after night worrying lest the oven might be too hot, or not hot enough for their gigantic culinary operations; while a haunting dread lest a cobweb might perchance be found in a remote corner of her home is responsible for many a woman seeking refuge in a "rest-cure" ward, where the crime of cobwebs—if there be any—will rest on other shoulders than her own.

Foremost among the causes producing rest-cure patients in the United States come "habits" of various kinds. And these patients are naturally the most difficult of all to deal with, for there is the mental and moral condition to combat, as well as the physical. There is the tea and coffee habit, which is common enough, into which the man or woman has insensibly fallen by allowing the health to run down. He may then easily yield to the temptation which naturally presents itself of a pick-me-up to sustain his energies sufficiently to get through the daily routine of life. Tea or coffee has been chosen perhaps with the idea that such stimulation is the least harmful "cocktail" to select. Once formed, the habit grows apace, and may be indulged in to such an extent that sleeplessness and nerve paroxysms threaten to undermine the whole nervous and mental system. These are cases just as difficult to nurse and care for as are the victims of the morphia, cocaine, or chloral habit, and all of these are unfortunately common enough in America.

### Mediæval Practice in the Care of the Sick.

### IV.—THE BLACK DEATH.

An outbreak of typhoid fever or small-pox nowadays is the signal for increased activity amongst sanitary inspectors and journalists; the sight of the Pardon graveyard full of the Black Death's victims led the *preux chevalier* Sir Walter de Mauny, and the then Bishop of London, to found the monastery in Charterhouse Square, in order that the sons of St. Bruno might pray for the souls of those who had perished without the consolations of religion. Every age has its own methods; the world improves, humanity progresses.

An old English metrical "Life of Saint Alexius" holds up the parents of the saint as a pattern of charity, in that they caused "all the sike of the borgh" to be brought to their house, "and to them to be good hede," and of "Seynt Briget" (patroness of the order, whose English nuns carried away to Lisborn on the accession of Elizabeth, and brought back into Devonshire, during the reign of Victoria, the keys of Zion House) it is related that "of her substance she repayred . . . many desolate hospytalls, and, as a busy administratrice, mercyful and pytous, she visited the nedy sycke men that were there, handled and washyd their sores without horror and lothesomnes."

Bishop Richard Poor, the probable author of the "Ancren Riwle," had considerable knowledge of medicine, and he took a practical, common sense view of illness, in spite of his love for mortification.

"Sickness," he says, "is a fire which is patiently to be en lured . . . sickness which God sends, but not that which some catch through their own folly. For many make themselves sick through their foolhardiness, and this displeaseth God."

Under the heading, "sickness which God sends," were classed, of course, the many frightful pestilences, like the Black Death and the "English Sweat," which ravaged the country, and frequently almost depopulated whole districts. It hardly becomes us, in the light of modern science, to find fault with our pious ancestors for so regarding the plagues, while we ourselves are still rather hazy in our knowledge of the influenza microbe, and the germ theory of disease has only so recently been demonstrated to be true.

An interesting book upon the Black Death has lately been published by F. A. Gasquet, D.D., O.S.B., in which he quotes from an old French manuscript to describe the labours of the nursing sisters in the Hôtel Dieu in Paris. The account he gives confirms an earlier one, in which Professor Hecker in his work, published by the Sydenham Society, speaks of "the faithful care of the sisters of charity, whose disinterested courage in this age of horror displayed the most beautiful traits of human virtue. For although they lost their lives, evidently from contagion, and their numbers were several times relieved, there was still no lack of fresh candidates, who, strangers to the unchristian fear of death, piously devoted themselves to their holy calling." Father Gasquet estimates the number of deaths among their patients at about 50 a day.

Whatever the causes and nature of mediæval epidemics in Europe, one thing is certain—that the supply of nurses was quite inadequate to meet the demands upon them.

In some places, for instance, the Black Death swept away a tenth of the whole people; there were no physicians to cure, nurses to tend, or priests to perform the last rites for the thousands of sick, abandoned to die amid surroundings which defy description. Boccaccio has given us an account of the Black Death in Florence. "When the evil had become universal, the hearts of all the inhabitants were closed to feelings of humanity. They fled from the sick and all that belonged to them, hoping by these means to save themselves. . . . Amid this general lamentation and woe, the influence and authority of every law, human and divine, vanished. Most of those who were in office had been carried off by the plague, or lay sick, or had lost so many members of their families that they were unable to attend to their duties. . . . Women, as well as men, abandoning their dwellings and their relations, retired into the country. But of these also, many were carried off, most of them alone, and deserted by all the world, themselves having previously set the example . . . one citizen fled from another—a neighbour from his neighbours—a relation from his relations; and in the end so completely had terror extinguished every kindlier feeling, that the brother forsook the brother—the sister, the sister—the wife, her husband; at last, even the parent his own offspring, and abandoned them, unvisited and unsoothed, to their fate. Those, therefore, that stood in need of assistance fell a prey to greedy attendants, who for an exorbitant recompense merely handed the sick their food and medicines, remained with them in their last moments, and then not unfrequently became themselves victims to their avarice, and lived not to enjoy their extorted gain."

These, fortunately, were not the only nurses, however. Professor Hecker, in his "Epidemics of the Middle Ages," pays frequent tribute to the virtue of what he terms "the charitable orders," who "did as much good as can be done by individual bodies in times of great misery and destruction"; and when the epidemic had sufficiently subsided to allow of reasonable enactments being passed to prevent a severe recurrence of the disorder, we hear of rules ordering priests "to examine the diseased, and point out to special commissioners the persons infected," while "those who attended upon a plague patient were to remain apart for ten days" before mixing with other people, and, moreover, "none except those who were appointed for that purpose were to attend plague-patients, under penalty of death and confiscation." Of course, such regulations were local, but charity was not confined to any country, and the need for organised nursing gradually led to the formation of religious orders devoted to the care of the sick.

### Appointments.

### MATRONS.

Lyme Regis Cottage Hospital.—Miss Adelaide Bourne has been appointed Matron of this hospital. She was trained at the Buckinghamshire General Infirmary at Aylesbury. She then took up private nursing for a time, and subsequently worked as district nurse in the parish of St. John's, Isle of Dogs, for four years. From December 15th, 1892, to October 23rd, 1894, she worked at St. John's Hospital for Diseases of the Skin, Leicester Square, W.C., and afterwards passed the examination of the London Obstetrical Society. Since then, until taking up her new appointment, she has been employed in Wilton Workhouse Infirmary.

Romney Marsh Union, New Romney, Kent.—Mrs. Mary Augusta Nelson (née Wimberley) has been appointed Matron of this institution. Mrs. Nelson was trained at the Mile End Infirmary, and has been charge nurse at the St. Pancras Infirmary: private nurse in connection with the Nursing Institute, Bournemouth; and superintendent of night nurses at Bethnal Green Infirmary.

Llandrindod Wells Hospital and Convalescent Home.—Miss Mary A. Evans has been appointed Sister-Matron at this institution. Miss Evans was trained at St. Mary's Hospital, Paddington, and for over nine years has held appointment as ward sister at that hospital. She has been for the last two years nursing private cases at Florence and Naples.

### The Ideal Nurse.

BY A MATRON.

"By love, serve."

"They must be persistent yet gentle, strong yet tender, with high ideal yet practical action, self-respecting yet unselfish, large in heart and rich in sympathy."

—*Knox Little.*

TOLSTOI says: "It appears that no one can lead a good upright spiritual life; the utmost people may do is to discourse about it." In the same way it appears no one can lead the life of an ideal nurse; but, at any rate, we can create her for the nonce and "discourse about her."

In the long list of virtues which must belong to this model creation—and yet after all she is to be a very human woman—it is difficult to say which is to have the pre-eminence. For equally indispensable are physical and moral strength, quietness and quickness, patience and unselfishness, sympathy and sense. Now these attributes are not opposed, but must be linked together, or they are shorn of half their power. For instance, sympathy may dwindle to mere weak sentiment (and do infinite harm to an hysterical patient) where it is dissociated from sound common sense. So, too, it is possible to be a quick worker, prompt in thought and action, without flying about like a whirlwind, and the perpetual centre of noise. A restful manner acts as a charm in imparting rest. But perhaps the most ideal qualities, because they are points of character, and thus lie deeper than manner, are, a divine patience, and a yet diviner selflessness. These, being in reality love—charity—are the very essence from which all truly nurse-like virtues spring.

It often seems like coming down from the mountain-top, after the contemplation of ethical beauty and perfection, to mix again in the prosaic trivialities of mundane existence. Yet it is in these prosaic trivialities (though they are not in reality trivial) that a nurse's duty lies; but she should raise these to a higher standard, and work her ideal into them. Even though it seems impossible of attainment, it is yet better to set our aspirations high, and to have an "ideal, without which our lives are barren and our hearts are dead," than to live without high aim "from craven fear of being great." All too little "greatness" comes into the lives of most of us, partly because, afraid of the failures which lie in its path, we are content with being very little indeed. Perhaps little-mindedness is one of the faults to which nurses are most prone in their somewhat narrow sphere of monotonous daily occupation, and living as they do in a community of women, where petty jealousies are apt to be rife. They sorely need more breadth and generosity in their dealings with one another; their little bickerings and assumptions of authority are beneath the dignity of women—especially when engaged in such holy work as nursing is, or should be.

But again, to speak of nursing as a "narrow sphere of monotonous daily occupation" is to convey a false impression. It may have a narrowing effect, if nothing beyond the mere drudgery is thought of; but, to the less superficial, it is deep and wide and very full of human interest. I should put this human interest very much to the fore: it far transcends the surgical or medical interest of the "case." This is sometimes apt to be lost sight of in hospital, where the press of work and the idea of system are often predominant. We should guard against becoming mere hospitalised sticks, the embodiment of a cold, formal routine; officials of an institution, and nothing more. System is an invaluable handmaid but a heartless master; it should guide, but not predominate. The "milk of human kindness" should never be frozen to the ice of formalism, for a kind heart can often do what a skilful hand cannot, and professionalism will never perform the work of sympathy. Yet we must not for a moment under-rate the value of methodical work and professional training, nor forget the necessity of discipline. Only, before even the imperative demands of work, certainly before any self-seeking on the part of the nurse, should come the comfort of the patient and gentle care for the sufferer, who is not only "No. 11," but a fellow human being. And the stories of the individual lives of each, add greatly to the interest of nursing them. Tragedy, comedy, or pathos are in them all—too often they are tales of sorrow. And what if it is "but one sad little story of all the heaped-up sorrow of the world," yet the nurse may help to lighten it. It is worth while. And how much courage, patience, even heroism is often found among even the "roughest customers"!

Speaking of nursing, as applied to hospital nursing, there is another point of duty to notice. This is the behaviour of nurses towards their superiors—for superiors in work all those in authority are; other details are beside the mark. Obedience to orders, respect and loyalty to those who give them, are the unquestioning duties of every nurse. They should always uphold the authority of the Sisters before the patients, allowing no adverse criticism in their hearing; and the sisters, in their turn, will support their subordinates. The patients should never see any jar in the harmony of those who are working for them; and considering what the work is, sisters and nurses, seniors and juniors, should always be working together.

Another thing to be remembered is that every unit is in a degree responsible for the whole; or, according to Spenser, the sociologist, "Nothing comes out of a society but what originates in the motive of an individual." So every nurse stands not alone, but represents her profession, and it is either exalted or degraded by her. The thought of this might prove a salutary restraint to those who really care for the honour of the nursing profession as a whole, but appear to care very little for their own conduct in particular. When we hear nurses spoken of as "a fast, giddy set of girls, absolutely thoughtless of their patients, and who have taken up nursing from worldly and unworthy motives," let us look to ourselves, and find the reason of such accusation. And again, when nurses are upheld as patterns of good, gentle, self-denying women, living noble lives, let us look within and see if we deserve the praise, or if we are hypocrites, selfish, and unchristian, though wearing the cloaks and veil of self-abnegation and devotion to the sick.

Such a weighty list of virtues may seem too heavy for an average woman. But the good nurse should not be bowed down by her responsibilities, or go about a poor depressed creature. Nowhere is cheerfulness more necessary than in the sick-room. If the nurse is not naturally happy-hearted, she must, at any rate, cultivate a bright manner. The invalid is so apt to be a dreary pessimist, and even to delight in his pessimism, that it is all-important the nurse should take an optimistic view of life in general, and of her patient's case in particular. Mind and body are so closely allied, and the mental so largely affects the physical nature that recovery may actually be promoted by hopefulness of spirit.

Then, even for the humblest worker, it is possible—without being obtrusively "goody"—to live Christ in the wards. That is, to live a practical, everyday life, as He did, yet with the stamp of a higher life upon it. And nurses have especial opportunity of "going about doing good." Though no good can be done unless the most "trivial" matters of ward work and duty are first scrupulously attended to. Conscientiousness is before all things necessary. The words addressed to the steward may be applied with special aptness to nurses: "Before all things it is required that a nurse be found faithful."

### A Family Home.

It is the boast of England that the words "family" and "home" go together. Yet often the home is broken up through the death of the bread-winner, or sometimes of the mother, and then the family drifts apart. How frequent is the record of accidents to children when the father is dead and the mother is compelled to go out to earn the living. They play in the street at the risk of being run over by every passing vehicle, or they are left at home and manage to burn themselves or fall out of window. At best they pass long dreary hours, lonely, often cold, and as often hungry. Even when the father is alive and earning a sufficient wage, if the mother be dead the lot of the children may be little better, left either to chance or to the care of indifferent and pre-occupied neighbours.

It is for the sake of such cases that the Glasgow Corporation has established what it calls a "Family Home." The intention of this is to provide a place where widows or widowers with children can lodge, and where the children will be taken care of when their parents are out at work. The home has been established barely a year, and is not yet wholly self-supporting, but the demand for the conveniences it offers is increasing as the bereaved among the working class come to know it and appreciate its worth, and there is every reason to believe that before long it will cease to be, in the narrowest sense, a charity, while it will remain in very sense a boon.

The Family Home contains 158 bedrooms, each intended to be occupied by one adult and two or three children. In detail, it's accommodation is as follows: on the ground floor are 23 bedrooms, the dimensions of which are 13 feet by 8 with a height of 12 feet. There is also a kitchen, 26 feet by 29; a large dining-hall, 32 feet by 60, lighted by a cupola; a women's sitting-room of the same size as the kitchen, and a children's hospital measuring 13 feet by 25. Besides these apartments the ground floor contains the superintendent's house and office, the store-rooms, wash-house, bath-rooms, and lavatories. A hydraulic lift to the upper floors fills up the remaining space. The second, third, and fourth floors are alike. Each contains 45 bedrooms, the same in floor space as those below, but a little lower in ceiling—10 feet 6 inches instead of 12 feet. There is also for each floor a large recreation-room, 26 feet by 46; a dining-room, 18 feet by 12½; and a kitchen, 12½ feet by 8. The fifth floor is given up to bedrooms, baths, and lavatories for the servants.

It will be noted that there is a kitchen on each floor. The large one on the ground floor is intended for the general cooking of the establishment. But as some of the lodgers prefer to buy their own food, they are allowed to do so and to cook it in the kitchen belonging to their floor. For those who take the meals prepared by the authorities breakfast is provided at a cost of 2½d., dinner at 4d., and supper at 3d. This is the rate for adults; board for children under 12 is at a cheaper rate, viz., for a single child, 1s. 10d. per week; but, when there are two children in one family, the charge is 1s. 7d. each; and, when three or more, 1s. 4d. each. It is a rule, however, that children's board and the rent of rooms must be paid weekly in advance. In face of these rates, it is satisfactory to record that the money received for meals fully covers the cost. The average number of meals sold is about 60 per day.

The bedrooms contain each an iron bed, an iron crib, a table, two chairs, a stool, a looking-glass, and a small chest of drawers. The rent varies with the sex of the adult occupant. Thus fathers pay 3s. 6d. a week and 8d. for each child; while mothers, though their children are charged at the same rate, pay only 2s. 6d. a week for a room. The reason of this is that the servants of the home have to clean the rooms occupied by men, while the women are expected to clean their own. The matron has the right of access to all rooms during the day to see that both do their work properly, and also to secure that rooms are properly aired.

Children are under the control of the matron during their parents' absence. There are resident nurses, who see that the elder children are dressed and sent to school, while the younger ones are looked after in the home. There is a yard attached to the building where they can play in fine weather, while the recreation-room on the top floor forms the children's day nursery. What a change from the lonely attic or the dirty, dangerous street.

In case of non-infectious illness among the children they are treated for a week in the hospital free of charge by the doctor who attends the home. After that their parents are expected to provide attendance and medicine for them. Infectious cases must, of course, be at once removed.

Of course, in return for these advantages there are some restrictions, though not onerous ones, on individual freedom. Residents are expected to be in the home by half-past ten, though by arrangement with the matron they may be allowed to stay till a later hour. At eleven o'clock all lights—they have electric light—must be turned out. It is forbidden to bring spirits into the home, and any unruly resident may be dismissed on forty-eight hours' notice. From any who go voluntarily the same notice is required.

In face of the ample accommodation and the various advantages, it may seem strange that as yet the largest number of residents has been 36 adults and 82 children. But the home has not been long started, and it takes ignorant people some time to learn the usefulness of any new institution. Some may think it akin to the workhouse; others whose self-respect may not hinder them from taking any advantage they can get, love too well their liberty—liberty to get drunk and come in at any hours they like. On the other hand the officials of the home must exercise a certain selection; the home is meant for the respectable working class, and disreputable and noisy members must be ejected. But the number of occupants is steadily increasing, and there is every appearance that the home is doing the work it was meant for, that it may help to keep families respectable that might otherwise drift into the streets, may enable widows to avoid the workhouses, and save widowers from those hasty and ill-considered marriages by which they try, often in vain, to solve the problem of bringing up their motherless children.

### Royal British Nurses' Association.

### BREAY v. THE ASSOCIATION.

On the 15th and 18th inst. the Court of Appeal heard the appeal against the interim injunction granted by Mr. Justice North on the 19th May restraining the Association from expending its funds in defending the action brought by Dr. Bedford Fenwick against Miss de Pledge. The appeal was allowed, the interim injunction being discharged, and Miss Breay being ordered to pay the costs of the motion in the Court of Appeal and the Court below.

### Presentations.

On Monday, June 21st, the nursing staff of the Borough Hospital, Mount Gould, Plymouth, presented Miss Marion Bailey with a handsome dressing bag. Miss Bailey is unfortunately compelled to resign her position as matron on account of ill-health, and her staff have expressed their affection and esteem by a most useful gift.

### Everybody's Opinion.

[Correspondence on all subjects is invited, but we cannot in any way be responsible for the opinions expressed by our correspondents. No communication can be entertained if the name and address of the correspondent is not given, or unless one side of the paper only is written on.]

### A QUESTION OF MAINTENANCE.

"District Nurse" writes: Can any readers of The Hospital tell me their experience as to what is a fair and comfortable allowance to ask per week for board for one nurse and one maid who does all the work and washing of the house. Is £1 per week too much; that is, to provide soap, starch, &c., and all little cleaning appliances for keeping house in order and replacing anything that was broken, such as globes of lamps, cups, glasses, &c., or what would be the right money to ask for per week? It is a manufacturing country town, vegetables, fruit, and fish dear.

### THE UNIFORM QUESTION.

"Pensioner" writes: Referring again to the vexed uniform question, I have been thinking that perhaps the editor of The Hospital would kindly intercede on the nurses' behalf (as he has always shown such a deep interest in our welfare) to H.R.H. the Prince of Wales or to our beloved Princess, that they would graciously consent to help us in the protection of our uniforms by registration or otherwise. The badge or medal, as suggested in a recent number of The Hospital, are very well for nurses who are attached to institutions. But what about those nurses who, like myself, "work on their own account"? All *bonâ fide* nurses feel something can be done, but what?

[It is, of course, impossible that their Royal Highnesses the Prince and Princess should arbitrate upon the vexed question of protection of the nurse's uniform, as is suggested by a correspondent. The solution of the difficulty will probably be evolved gradually by those who feel it, and we shall be glad to ventilate any practical proposition. After all, the institutions who train nurses of all kinds should realise that their responsibility does not end when the nurse goes forth to take her place amongst the world's workers, and we feel that they should be moving in this matter.—Ed. *T.H.*]

### FEVER NURSING.

"Anonymous" writes: Would you allow me a short space in The Hospital to make a few remarks on the above subject, as latterly I have had that department of nursing brought to my mind by words passed upon it which are certainly not calculated to raise the idea of fever work, and being myself a trained fever nurse, I felt I would like to say a little in its favour. Being an enthusiastic reader of The Hospital, I noticed some time back a paragraph in the "Nursing Mirror" bearing upon a remark of some individual to this effect, "Oh, anyone will do for a fever nurse!" which remark was recalled to me latterly by one similar, viz., "Oh, So-and-so is fever nursing; what monotonous work." Now I wish to say that I fancy it has been fully proved of late years that "Anyone will *not* do for a fever nurse," and it is a great mistake that such a branch should be called monotonous and that fever work and nurses are so often spoken of disparagingly. It arises from the fact that so many are disposed to hold the opinion that there is comparatively nothing to learn in infectious nursing. May I be permitted to say to the contrary, that such work requires special skill and special training to be efficiently done, and that there is as much, if not more, real nursing in fevers than in any other department of the profession. Every would-be probationer to-day is desirous of general training; a fever hospital is not thought worth entering. But once to enter such an institution, and what a vast amount of training in real nursing is there; more than in the mechanical bandaging of limbs (with all respect to surgical work). The handling of fever cases is alone an art; then the feeding, watching, tending—all splendid training. Gentleness unlimited, tact, and nerve are all required in the good fever nurse, and in these days the passing of special examinations is necessary also. With regard to knowledge, it is my opinion that the trained fever nurse is quite capable of taking charge of a medical ward in a general hospital. Almost all the medical cases found in general wards arise as complications following the fevers in infectious hospitals, and, with regard to surgery, I myself, during my training, had excellent work in the pyemia and erysipelas wards. Much credit is due to the nurse in infectious work, as much is entirely left to her. Therefore, let no one speak lightly of fever nursing. It is, after all, the wiser plan to make yourself master of one department thoroughly than to have, as is often the case nowadays in hospital, a year's training and a scattering of a little piece of everything. Fever nursing is anything but monotonous. I have for one always found it most happy, enjoyable, and encouraging work.

### The Book World for Women and Nurses.

[We invite Correspondence, Criticism, Enquiries, and Notes on Books likely to interest Women and Nurses. Address, Editor, The Hospital (Nurses' Book World), 28 & 29, Southampton Street, Strand, London, W.C.]

Elementary Bandaging and Surgical Dressing, with Directions Concerning the Immediate Treatment of Cases of Emergency. By Walter Pye, F.R.C.S., late Surgeon of St. Mary's Hospital. Revised and in part re-written by G. Bellingham Smith, F.R.C.S., Surgical Registrar, Guy's Hospital. Seventh edition. (John Wright and Co., Bristol. Kegan Paul, Trench, Trubner, and Co., Limited. St. John Ambulance Association, St. John's Gate, E.C. 1896. Ninety illustration, 218 pages.)

This little book, having reached its seventh edition, is too popular to need any re-introduction to those for whom it is intended. A book needs be good in the first instance to bear revision and additions as well, just as these need to be good if they are to be a solid improvement to the book. Our thanks are due to Mr. Bellingham Smith for his manner of dealing with this one so as to bring it fully up to date, such a procedure being absolutely necessary in view of the many advances in surgical practice during the last ten years. The revision has been most carefully and completely done, the book remains an old friend in whose survival we rejoice, and we find it as reliable and useful as ever.

Willing's British and Irish Press Guide, 1897. (London: James Willing.)

The twenty-fourth annual of Willing's Press Guide is now presented to the public in a revised and extended form, though the price (1s.), be it observed, remains the same. A vast amount of reliable information is contained in the compass of this handy volume, which for reliability, information, and completeness stands alone of its class. The contents of the volume, of which space forbids a detailed account, includes an alphabetical list of newspapers and periodicals, provincial newspapers, London addresses of colonial and foreign newspapers, Continental newspapers, reporting and telegraphing news agencies, &c.

### Death in Our Ranks.

We regret to note the sudden death of Miss Evans, matron of Hinckley Cottage Hospital, from pleuro-pneumonia, on the 21st inst.

### Wants and Workers.

Nurse H., Cottage Hospital, Milton Abbas, Dorsetshire, asks if any reader of The Hospital will give her a bath chair for the cottage hospital and the district? Nurse H. has a number of very aged people under her care, crippled with rheumatism and quite unable to walk, to whom a bath chair would be the greatest boon, allowing them to take advantage of bright days to get out once more into the fresh air.

### Snakes in the Sick Room.

It is by no means unusual for a sick person, in one sense of the word, to "see snakes." But Dr. Arthur Stradling, whose life investigation of snakedom has thrown such new light on serpentine subtleties, has made a series of most interesting experiments on snake-venom as a digestive agent—for which useful purpose he believes Nature chiefly intended it, though, as he quaintly puts it, he "by no means underrates its ammunitional value." The venom represents the saliva of the snake, and a small quantity placed in a basin in contact with white of egg will speedily convince the most sceptical that it possesses the power to digest and dissolve albumen. Dr. Stradling, in his scientific investigations as to the stomachic value of snake venom, has repeatedly administered it to himself in duly digestive doses. "A small bird, even though extremely old and tough, may be rendered readily digestible by impregnation with snake venom, which softens the hard flesh," he says, quite as if this substance were readily at culinary command. He has frequently fed his pets, whose number is legion, when ill, on fowl and flesh previously soaked in venom, and is well pleased with its success in sick dietary.

Indeed, he found that the anguish experienced after food of any kind by a delightful but dyspeptic old toad was relieved only by a free mixture with his meals of fresh venom, and of this Dr. Stradling's collection of snakes ensures a large and unadulterated supply. The toad in question, whose venerable years had weakened the forces of his digestive fluids, had long been wont to regard a plump mouse as an indulgence belonging only to the days of his reckless youth. And the envious looks he cast on other more fortunate toads who could swallow so dainty a morsel without an after pang were most pathetic. Indeed, even the snake-heart might have been so touched by his distress as to voluntarily offer up the contents of his fangs so that a mouse-meal might be possible to a toad stomach enervated by a long and laborious life. The prospect of the lion lying down with the lamb is a mild hyperbole compared with the suggestion that snakes through the centuries have been yielding an obvious digestant for the use of the dyspeptic, the while bearing without reproach the suspicion that they were manufacturing a fluid beside which nitro-glycerine seems ideally harmless.

The use of venom in the cause of the invalid and the dyspeptic opens up a dozen new speculations in snake industry, and patients might even "see snakes" in their bed-rooms without incurring base suspicion, while the advance of science may lead to the establishment of Snakeries for the cultivation of a fluid whose properties are doubly valuable in these days of dyspepsia. To the coming race it may seem no novelty to find on their restaurant tables bottles of cobra sauce wherewith to mitigate the evils of a hard beefsteak or a tough spring chicken. And the acme of Arcadia should be reached by the pre-digestion of infant foods through a judicious use of the saliva of the snake.

All the pepsins, and most of the artificial digestive agents, are at present derived from animal secretions, and the addition of this valuable venom to the list would surely do much to break down the enmity existing between man and snakes since the beginning of time. The dyspeptic at any rate might feel some gratitude for dainties enjoyed through the good offices of philanthropic serpents. The evolution of snake venom is attracting much scientific attention at the present time. For it is held by some students of snake lore that at one period in the evolution of the snake its "venom" was harmless, and that it has only by slow degrees taken on those poisonous properties with which it will always be associated in the popular mind, even though it possess at the same time a valuable peptonising quality.

### Sketches of Hospital Life.

### THE BLIND PHILOSOPHER.

The blind philosopher, as he styled himself, earned a living in our district by singing and "discoursing." He was a handsome old man, with long silvery hair and beard. He was led about by a terrier, and had a tin mug hooked to his coat, into which admirers of the picturesque dropped their coppers. His singing was vile, but his "philosophy" was at least amusing. For a man who was blind and could not read the newspapers his knowledge of topics of the day was remarkable. Some people held that his blindness was a pretence, a view which might have had its source in a facetious paragraph which appeared in the local newspaper, describing a vision of a "blind" orator and his dog chasing a small boy with a tin mug along the canal bank. I remember meeting him one Saturday night shortly after this paragraph appeared. He was standing in the glare of light coming from the windows of a large draper's shop. A rapt crowd was in attendance, not listening to his declamation, but watching the motions of a drunken soldier, who, standing on the pavement in front of him, was executing sword passes with his cane. The metal point of the stick whizzed continually past within an inch of the orator's face, but he never flinched. There was something unpleasant in the scene, but it proved the old man was no humbug.

One of his favourite stands was just outside the hospital gates. He was singing at this spot one day when Mr. Q——, our oculist, drove up. He was an Irishman, kindly and impulsive. "Blind, my man?" he asked as the philosopher finished his song. "Yes, sir," was the answer. "Strange!" said Mr. Q——, looking at him more closely; then, after a moment's hesitation, "Look here, my man, I am an eye doctor, and am about to see my out-patients. Come downstairs; I may be able to do something for you. Sam!" to the porter, "take this man to my room." The philosopher was grateful, but shy. He delivered himself oracularly on the inevitableness of fate and the wisdom of bowing to its decrees. "That's common sense," said Sam. "If you've got to go downstairs, you've got to," and marched him off to the consulting-room, where I was waiting with a ward case. "*Quite* blind?" asked Mr. Q——, in a tone of slight surprise, as he examined a pupil by the light of the window. "Quite," said the patient, with a sigh. "Nurse," said Mr. Q—— to me, "please take this man to the dark-room." I led the philosopher away, his dog following. The dark-room was a wooden compartment fitted up in the corner of a wide passage, into which a door from the consulting-room led. "What's he going to do?" asked the patient with more trepidation than one would expect from a philosopher. "Mr. Q——," I explained, "is going to throw a light into your eyes so that he can see exactly what is wrong with them." The philosopher groaned and muttered something in an undertone. "What did you say?" I asked. "Nothing," he answered—then, peevishly, "The doctor wants you. He's calling." The blind are remarkably acute of hearing. I found Mr. Q—— talking over the ward case with the house surgeon, and my name had never been mentioned. A few seconds later I returned with the doctor to the dark-room. It was empty. The passage led into a little square yard, the exit from which was locked. On one side it was bounded by a wall about four feet high, with a drop of five feet into a huge woodyard intersected by railway lines. Near this wall I found a penny. "That's out of his mug," said Mr. Q——, with a twinkle in his eye. "He's gone over the wall."—J. B. and M.D.

### "THE HOSPITAL" CONVALESCENT FUND.

We acknowledge with thanks a gift of 2s. 6d. to "The Hospital Convalescent Fund" from Nurse Frances.

### Eggs—Nurses.

We are all familiar with the classification of eggs—new-laid eggs—fresh eggs—eggs. It has occurred to me that nurses might be classified in a somewhat similar manner—born nurses—made nurses—nurses.

Born nurses are rare commodities, but we occasionally come across women whose whole appearance, demeanour, and character betoken their innate suitability for tending the sick. They bear the unmistakable impress of high moral rectitude and aptitude for work entailing self-denial and self-sacrifice. From the early dawn of maturity they have recognised their vocation, and have planned their lives to gain the training without which even the born nurse must fail. There are plenty of spurious born nurses. In fact it at once arouses suspicion to hear the mother of a would-be probationer exclaim, "Oh, but my daughter is a born nurse; our family doctor says so." She may be one of the rare ones, but we do not take it on trust.

Then there is that larger class which consists of made nurses. Large-minded and capable women, who in early life never supposed they would have to work for their living, and who are awakened later on to the fact that for some reason they must find a definite occupation. They turn to the nursing profession because it is a womanly one, because it presents a high ideal, and because there is an outlet for their ambitions. They seek a first-class training, they put forth their best energies, and by their determination to succeed, and by the sheer force of their personality they become excellent nurses, and obtain very often the highest appointments of their profession.

Then we come to the staple article—nurses. Of these there are countless grades, and they form the bulk of the nursing world. Some are good, some middling, others useless. Some there are who do their best and succeed well in a limited sphere, bright examples often of patience, perseverance, and industry. Others are content with mediocrity—they do their work just because it happens to be their work; they have no ideal, and jog along indifferently with few ambitions beyond their daily bread. Others, again, prove by their incompetence that they have mistaken their vocation.

There is yet another class of egg—one which is used only for electioneering purposes—rotten. No doubt there is this equivalent also in the nursing world, but, thank God, it is but seldom we are shocked by the proof of absolute worthness in our ranks.

Moral. We are not all born nurses. We cannot all of us be amongst those who, by their exceptional ability and strong individuality, win for themselves the highest posts. There are none, however, who may not strive to be amongst the foremost of the rank and file. All can try to raise the tone of the profession and prevent themselves from lapsing into inferiority. There is scope for all, and a field of work where each toiler may reap the fruit of her labour, even in this thankless and ungrateful world.

### Minor Appointments.

Union Hospital, Newcastle-on-Tyne.—Miss Annie Hedley has been appointed to the post of Charge Nurse at this institution. Miss Hedley received her training (three years) at the Royal Infirmary, Newcastle-on-Tyne, and since May, 1896, has held the appointment of head nurse at the Cancer Hospital (Free), Brompton. Her testimonials are good.

St. Olave's Infirmary, Rotherhithe.—Miss Florence Capes has been appointed Charge Nurse at this infirmary. Miss Capes received three years' training at St. George's Infirmary, Hanover Square. Subsequently she was appointed nurse at the Banstead Road School, where, in which capacity, she remained for three years. Miss Capes has also had training in fever nursing at the Stockwell Fever Hospital. She holds good testimonials.

### Notes and Queries.

The contents of the Editor's Letter-box have now reached such unwieldy proportions that it has become necessary to establish a hard and fast rule regarding Answers to Correspondents. In future, all questions requiring replies will continue to be answered in this column without any fee. If an answer is required by letter, a fee of half-a-crown must be enclosed with the note containing the enquiry. We are always pleased to help our numerous correspondents to the fullest extent, and we can trust them to sympathise in the overwhelming amount of writing which makes the new rules a necessity. Every communication must be accompanied by the writer's name and address, otherwise it will receive no attention.

*Nightingale Uniform.*

(300) Can anyone tell Nurse Gertrude where the grey alpaca which used to be the uniform of the Nightingale nurses can be procured?

Perhaps some of our readers can give Nurse Gertrude the information she requires. She would, however, certainly be able to find out from Miss Gordon, at St. Thomas's Hospital.

*A Medical Question.*

(301) Mrs. R. asks advice as to the treatment of one "suffering from growth connected with the kidney" and complications.

We can only recommend that a doctor be called in at once.

*The Midwives' Bill.*

(302) Having practised for eighteen years can I legally style myself a midwife, or must I send in my claim to the register, not having a certificate? I can have testimonials from doctors.—*Mrs. D.*

Unfortunately at present there is nothing to prevent any woman styling herself midwife. If the Bill now before Parliament passes into law you will then have to send in your claims to be placed on the register. Any such register will allow of the inclusion of a certain number of those women who have been acting in the capacity of midwife previous to the passing of the Act.

*Patient Wanted.*

(303) Will you kindly advise me as to the best means of obtaining a patient mentally deficient?—*A Constant Reader.*

You should make your wish to undertake the care of a patient known as widely as possible amongst your acquaintances. To do this and to advertise are the only ways of getting what you want that we can suggest.

*Oxygen Treatment.*

(304) Can you tell me whether the new "Oxygen Treatment" can be administered privately or only in hospital? If the former, who is the best person to whom to apply?—*Inquirer.*

Consult your medical man on the subject.

*Midwifery.*

(305) Kindly tell me if Part XI. of Witkowski's "Human Anatomy and Physiology" would be of use in studying midwifery?—*M.*

Any handbook of human anatomy in which the pelvic organs are described will, so far as that goes, be of use, but the anatomists enter into details which are not required by and would often only puzzle midwives. Have you studied Fly Smith's "Handbook for Midwives" or Herman's "First Lines in Midwifery"?

*Home Wanted.*

(306) Can you recommend a home where a poor gentleman subject to occasional fits could be taken in?—*Enquirer.*

You will find lists of homes and institutions in Burdett's "Hospitals and Charities" (Scientific Press, 28 & 29, Southampton Street, Strand, W.C.) Why not apply at the Hospital for Epilepsy and Paralysis, 32, Portland Terrace, Regent's Park, N.W.

*A Nurse and "The Hospital."*

(307) Will you please tell me the name of the nurses' association whose members can obtain The Hospital for 1d. weekly? I wish to join the association.—*F. L.*

You can get the "Nursing Mirror" at a cost of 1d. weekly from the Office of The Hospital, or by a yearly subscription of 6s. 6d. If by the "nurses' association" you mean the Royal National Pension Fund for Nurses, write to the Secretary, 28, Finsbury Pavement, E.C.

*Monthly Nursing.*

(308) I have been trained in monthly nursing and have got a certificate. How can I get cases? I should prefer to work in London.—*Perplexed.*

Consult the matron at the hospital where you trained. You should endeavour to obtain introductions to doctors in the neighbourhood in which you think of settling. If you belong to the Trained Nurses' Club and Midwives' Institute you can enter your name on the register of the institute. The lying-in hospitals keep registers for the midwives and nurses they have trained.

### Where to Go.

The Working Ladies' Guild.—The summer sale of the Working Ladies' Guild will be held on June 30th and two following days at 1, Belgrave Square. H.R.H. Princess Frederick will open the proceedings on June 30th. Doors open from two to seven. The Jubilee present to her Majesty the Queen by the committees of the Working Ladies' Guild, worked and painted in the departments, will be on view during the sale.

